# Temperature-humidity evolution and radon exhalation mechanism of red clay-bentonite covering layer in uranium mill tailings pond

**DOI:** 10.1038/s41598-023-50733-w

**Published:** 2024-01-30

**Authors:** Chao Xie, Wenjun Lu, Hong Wang, Xiangshuai Wang, Tao Yu

**Affiliations:** 1 School of Nuclear Science and Technology, University of South China, Hengyang, 421001 China; 2 School of Resources, Environmental and Safety Engineering, University of South China, Hengyang, 421001 China; 3 Key Laboratory of Advanced Nuclear Energy Technology Design and Safety Ministry of Education, University of South China, Hengyang, 421001 China

**Keywords:** Environmental impact, Nuclear waste

## Abstract

To ensure the safety and stability of the beach surface of the decommissioned uranium mill tailings pond, this paper uses red clay-bentonite and red clay (1:1) to carry out covering layer radon reduction simulation experiments to study the temperature, humidity, and radon reduction effect of the covering layer under natural conditions. The results show that the radon exhalation rate of red clay-bentonite cover layer is only 0.32 times that of red clay, which has a better radon reduction effect. The red clay-bentonite cover layer has better water retention and comparable heat preservation effect than red clay cover layer. The red clay-bentonite and red clay temperature curves follow the same evolution trend and were close together in the same outdoor conditions, and the humidity curves showed a difference of 1% to 3%. Soil temperature is the dominant factor affecting the variation of radon exhalation of red clay-bentonite and red clay covering layer with unsaturated water content.

## Introduction

Uranium mill tailings (UMTs) pond is a nuclear facility for storing uranium tailings. Radon (^222^Rn) is a decay product of radium contained in tailings, which is a radioactive gaseous nuclide. Radon can migrate with water and air in the geological environment of UMTs ponds. Radon can enter the human body through respiration and induce lung cancer by internal irradiation^1^. Therefore, radon is an important hazard source threatening the health and safety of residents around the UMTs pond.

Covered disposal is one of the most effective ways to reduce radon exhalation from radioactive solid waste^2^. At present, covering disposal is the most common method of beach surface disposal in the decommissioning UMTs ponds. Scholars have studied the radon reduction performance of different covering materials, such as waste rock, sandy soil, laterite, kaolin, sand- mixed laterite, and waste rock-laterite mixture, etc.^3–6^. The radon reduction performance of the cover layer is closely related to material properties and cover parameters, including material composition, porosity, water content, particle size distribution, compaction, cover thickness, etc.^7–9^. The radon reduction effect increases with increasing soil thickness and compaction^10,11^. Multilayer covering also has significant influence on radon control performance of UMTs beach surface^7^. Under the same thickness conditions, Radon reduction coefficient increases with the increasing fractal dimension of the size distribution of covering material^3^. Radon exhalation can be effectively reduced by using low-permeability covering material^12,13^. The essence of radon reduction by covering is to keep radon in the covering layer as much as possible and let it decay naturally to achieve the purpose of reducing radon exhalation from the covering layer. Bentonite has good physical properties and is often used as the covering material of UMTs ponds^4,5^.

Bentonite is a natural resource with abundant storage and relatively low prices. Bentonite contains mainly the mineral montmorillonite ((Na.Ca)_0.33_(Al.Mg)_2_[Si_4_O_10_](OH)_2_·nH_2_O) and is commonly used as an adsorbent and clay modifier. Montmorillonite increases the total specific surface area and cation exchange properties of clay soil, resulting in high moisture absorption and low permeability of soil^14,15^. Adding sodium bentonite to soil can increase soil strength and decrease soil permeability effectively^16^. The soil is modified by adding bentonite, which can fill or plug the soil pores and transform the large pores into small ones^17^, to improve the mechanical properties and impermeability of the modified soil^18,19^. The decreasing porosity and permeability of the cover layer can strengthen the inhibiting effect of the cover layer on radon exhalation. Therefore, it is feasible to modify the clay soil cover with bentonite to improve its radon reduction effect. Considering the economy and practicability of covered disposal, bentonite is often mixed into soil for local soil modification to improve soil performance.

At present, there are many studies on the structure and properties of bentonite-modified soil, but the law and mechanism of radon reduction by bentonite-modified soil are still unclear. The response characteristics of bentonite-modified soil covering layer to the natural environment also need to be further studied. In this paper, the red clay and the red clay-bentonite mixture are used as the covering material respectively to carry out the laboratory simulation test for the treatment of UMTs pond beach surface. By analyzing the radon precipitation rate, temperature and humidity of the cover layer, the radon control performance and mechanism of bentonite modified cover layer will be obtained. The research can provide a basis and reference for the implementation of decommissioning and treatment of UMTs ponds.

## Materials and test methods

### Test materials

The UMTs used in the test are taken from a UMTs pond in southern China. Its covered material is red clay, and calcium bentonite is an additive. The mineral composition of the materials is measured by ZSX Primus II X-ray fluorescence spectrometer of RIGAKU, Japan, and the results are shown in Table 1. According to the Standard for geotechnical testing method GB/T 50123-2019 measures the basic physical and mechanical properties of the red soil. The LP-100D digital soil liquid plastic limit tester is used to measure the liquid plastic limit of red soil. The basic physical and mechanical properties of the red clay soil are shown in Table 2.

**Table 1:** Mineral composition of the material (%).

Material	SiO_2_	Al_2_O_3_	Fe_2_O_3_	MgO	CaO	K_2_O	Na_2_O	TiO_2_	Others
UMTs	84.71	7.41	2.2	0.12	0.28	3.47	0.42	0.38	1.01
Red clay	51.67	16.50	8.52	2.48	14.08	4.02	0.56	1.58	0.59
Calcium bentonite	70.65	16.40	4.71	1.52	5.20	0.44	0.40	0.67	0.01

**Table 2:** Basic physical properties of red clay.

Specific gravity	Maximum dry density (g·cm^−3^)	Optimum moisture content (%)	Plastic limit (%)	10 mm liquid limit (%)	17 mm liquid limit (%)
2.5	1.9	23.3	26.3	42.8	50.3

### Experimental device

**Figure 1:**
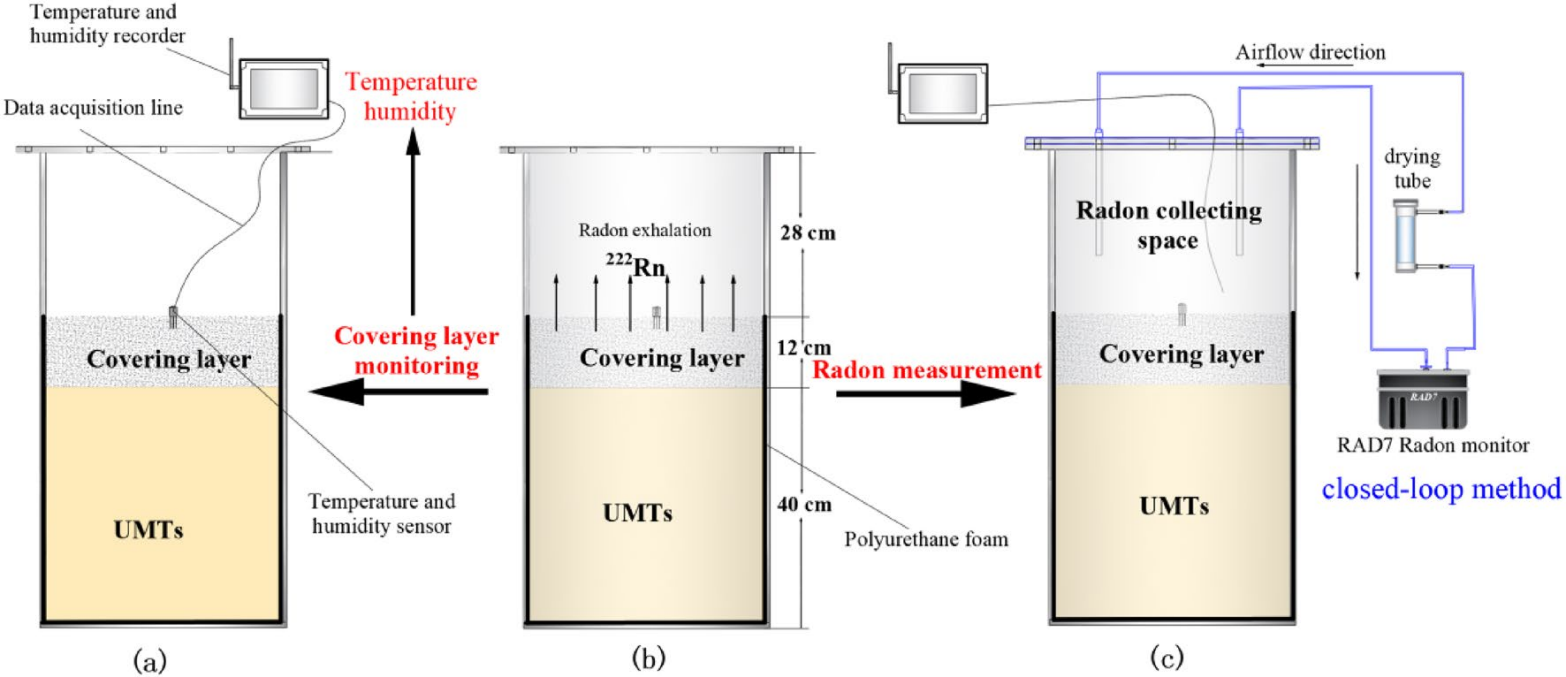
Experimental flow and experimental device diagram.

The experimental device mainly includes an experimental model chamber, a parameter monitoring device and a radon measurement part. As shown in Fig. 1, the main body of the experimental model chamber is a sealable cube box with a size of 40 cm × 40 cm × 80 cm, which is mainly used for placing the model of UMTs pond beach surface. The lower and bottom walls of the box are equipped with a polyurethane foam insulation layer to reduce the influence of ambient temperature on the UMTs. When the chamber is sealed, the upper space of the chamber can be used as a radon-collecting space (Fig. 1c), and the radon exhalation rate of the covering layer can be measured by the closed-loop method^6,20^.

Radon measurements were carried out using a RAD7 radon monitor, which had been calibrated in the Radon Laboratory of the University of South China before the experiment. The Ubibot GS1 industrial temperature and humidity recorder was used for real-time continuous monitoring of temperature and humidity as shown in Fig. 1a. The measuring range of temperature and humidity is 20 °C–60 °C and 10–90% respectively, and the accuracy is ± 0.5 °C and ± 2% respectively.

### Experimental program

Two covering beach surface models of UMTs pond were prepared and placed in different experimental model chambers respectively. The experimental models were covered by red soil and clay-bentonite respectively. Red clay covering layer (RC) was the red clay used to cover UMTs. The red clay-bentonite covering layer (RC-B) was a mixture of 1:1 red clay and sodium bentonite to cover UMTs. The thickness of the UMTs and covering layer was 40 cm and 12 cm respectively, and the height of the radon collection space was 28 cm.

The models were prepared by layer-loading method. The UMTs were filled in four times, each filling thickness was about 10 cm. The cover layer was filled twice. After preparation, the two groups of experimental models were placed in the same natural environment for 7 days.The temperature and humidity recorder was used to continuously take the temperature and humidity of the covering layer. The radon data acquisition of the two models adopted the same time measurement. Radon concentration data was sampled at an interval of 10 min and each sampling lasted 3 h. We collected five sets of radon concentration data and extracted temperature and humidity data at the same time. These 5 measuring periods were numbered as measurement points 1 to 5.

### Calculation method of radon exhalation rate

The closed-loop radon measurement method is the most used one to measure the radon exhalation rate. The mathematical model for the increase of radon concentration in a concentrated radon cover is as follows^21,22^:

1 $$ \frac{dC}{{dt}} = \frac{JS}{V} - \lambda C - \zeta_{k} C - \zeta_{x} C, $$

where C is the concentration of radon accumulated during time, Bq·m^−3^; J is the radon exhalation rate, Bq·m^−2^·s^−1^; S is the measured area, m^2^; V is the volume of the radon collector, m^3^; t is the unit time, s; λ is the radioactive decay constant of radon (2.1 × 10^−6^ s^−1^); $\zeta_{k}$ and $\zeta_{x}$ are the coefficients associated with radon leakage and anti-diffusion, s^−1^. Anti-diffusion refers to the diffusion of the radon in the radon collector back into the medium.

When $\left. C \right|_{t = 0} = C_{0}$ (That is, the initial radon concentration is *C*_0_, Bq·m^−3^), Eq. (1) can be solved as follows:

2 $$ J = \frac{{\left( {\lambda + \zeta_{k} + \zeta_{x} } \right)V}}{{S\left[ {1 - {\text{e}}^{{ - \left( {\lambda + \zeta_{k} + \zeta_{x} } \right)t}} } \right]}}\left[ {C - C_{0} {\text{e}}^{{ - \left( {\lambda + \zeta_{k} + \zeta_{x} } \right)t}} } \right]. $$

For the convenience of calculation, the calculation formula for radon exhalation rate is often simplified. Table 3 lists the simplified calculation formula of radon exhalation rate and the conditions required for adopting this method. The formula $J = kH$ is used to calculate the radon exhalation rate in this paper.

**Table 3:** Simplified formulas and conditions for calculating radon exhalation rate by the closed chamber method.

Simplified formula	Conditions to be met	
$J = \frac{\lambda V}{{S\left( {1 - {\text{e}}^{{ - \lambda {\text{t}}}} } \right)}}\left( {C - C_{0} {\text{e}}^{{ - \lambda {\text{t}}}} } \right)$	(1)	The radon leakage and anti-diffusion are ignored
$J = \frac{{V\left( {C - C_{0} } \right)}}{St}$	(2)	① Meet condition (1) ② $\lambda t < < 1$
$J = \frac{Vk}{S}$	(3)	① Meet condition (2) ② The cumulative radon concentration is linear with the cumulative time, and *k* is the slope obtained by using the least square method, Bq·m^−3^·s^−1^
$J = kH$	(4)	① Meet condition (3) ② The radon exhalation surface is consistent with the base area of the radon collector, and the radon collector is cylindrical or cubic. H is the height of the radon collector, m

## Results and discussion

### Analysis of cover temperature and humidity

**Figure 2:**
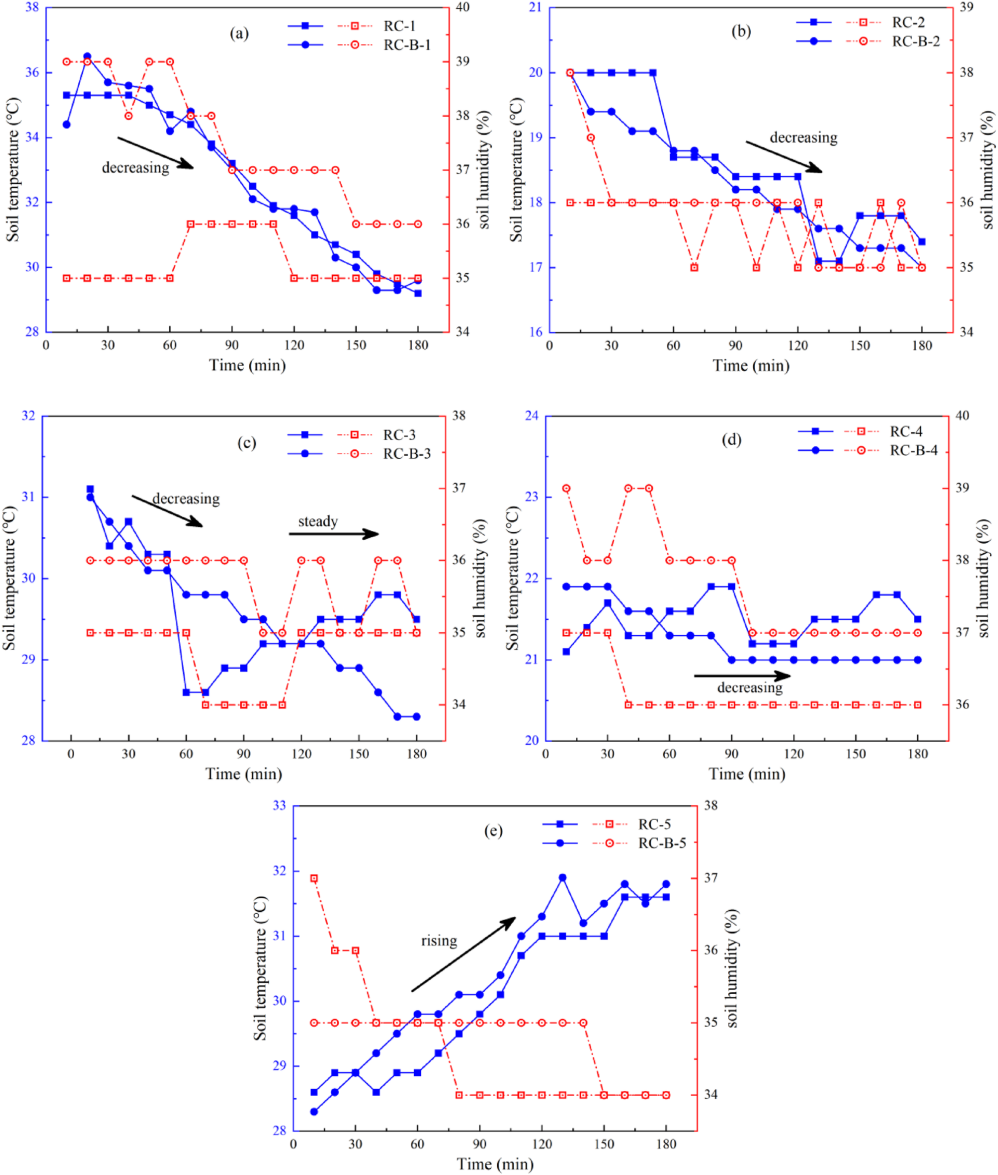
Evolution during time of temperature and humidity in the beach covering layer at different measurement points ((**a**–**e**) measurement points 1–5).

Figure 2 shows the temperature and humidity evolution of the covering layer in different outdoor periods. There was no rainfall during the 5 measurement periods, and the temperature and humidity of the covering layer were mainly affected by the outdoor ambient temperature and sunshine. In Fig. 2, the temperature variation intervals and trends of RC and RC-B in different measurement periods are obviously different, but the temperature curves of the two during the same measurement period show obvious overlap. This indicates that the outdoor ambient has a similar effect on the temperature of RC and RC-B, and the bentonite cannot effectively improve the thermal insulation performance of the red clay covering layer.

In Fig. 2a, the temperature curves of RC and RC-B show a decreasing trend within a wide range of 29.2–36.5 °C, and the humidity curves are obviously separated. The RC humidity curve remained in the range of 35.0–36.0%, and the RC-B humidity curve decreased from 39 to 36%. In Fig. 2b, the temperature curves show a decreasing trend in the small range of 17.2–20.0 °C, and the values of the RC-B humidity curve are about 1% higher than that of the RC humidity curve. In Fig. 2c, the temperature curves show a decreasing-steady trend within a small range of 28.2–31.1 °C, and the values of the RC-B humidity curve are about 1–2% higher than that of the RC humidity curve. In Fig. 2a–c, the temperature curves show different decreasing trends, but the values of RC-B humidity curves are all greater than those of RC humidity curves.

In Fig. 2d, the temperature curves of RC and RC-B show a steady trend in the range of 21.1–21.9 °C, and the values of the RC-B humidity curve are about 1–3% higher than that of the RC humidity curve. In Fig. 2e, the temperature curves showed a rising trend within a small range of 28.3–31.1 °C, and the humidity curves of RC and RC-B showed a cross phenomenon. The RC-B humidity change is not significant, and the RC humidity curve shows a significant decreasing trend. These phenomena indicate that there are significant differences in water retention performance between RC and RC-B, and RC-B has better water retention performance.

### Analysis of radon exhalation characteristics

**Figure 3:**
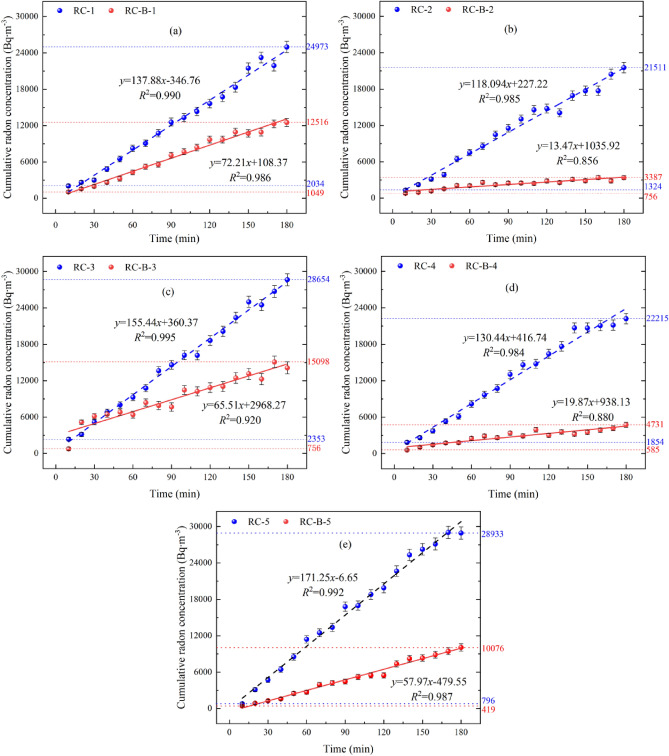
Cumulative values of radon concentration on the beach covering layer at different time measurement points (a-e measurement points 1–5).

The cumulative radon concentrations in the RC and RC-B beach covering layers are shown in Fig. 3. The cumulative radon concentrations during time at the five different points show a good linearity according to R^2^ values ranging from 0.856 to 0.995. The maximum values of radon concentration from RC at 180 min were about 24,973 Bq∙m^−3^, 21,511 Bq∙m^−3^, 28,654 Bq∙m^−3^, 22,215 Bq∙m^−3^, and 28,933 Bq∙m^−3^. Those from RC-B at 180 min were about 12,516 Bq∙m^-3^, 3387 Bq∙m^−3^, 15,098 Bq∙m^−3^, 4731 Bq∙m^−3^, and 10,076 Bq∙m^−3^, respectively. The radon concentration in the collector exhalation from RC covering is significantly higher than that of exhalation from RC-B covering.

The slope of the fitted curve of cumulative radon concentration, *k* (Bq∙m^−3^∙min^−1^), indicates the rate of increase of radon per unit time. The radon exhalation rate from RC-B is significantly lower than that from RC, and the RC-B had a significant radon reduction advantage. Deng et al.^23^ carried out an experiment with soil improved by bentonite to reduce the radon exhalation and arrived at the same conclusions. After the 40 cm thick soil had been improved by bentonite, the radon exhalation rate was reduced from 0.23 Bq∙m^−2^∙s^−1^ to 0.11 Bq∙m^−2^∙s^−1^.

### Analysis of radon reduction effect

**Figure 4:**
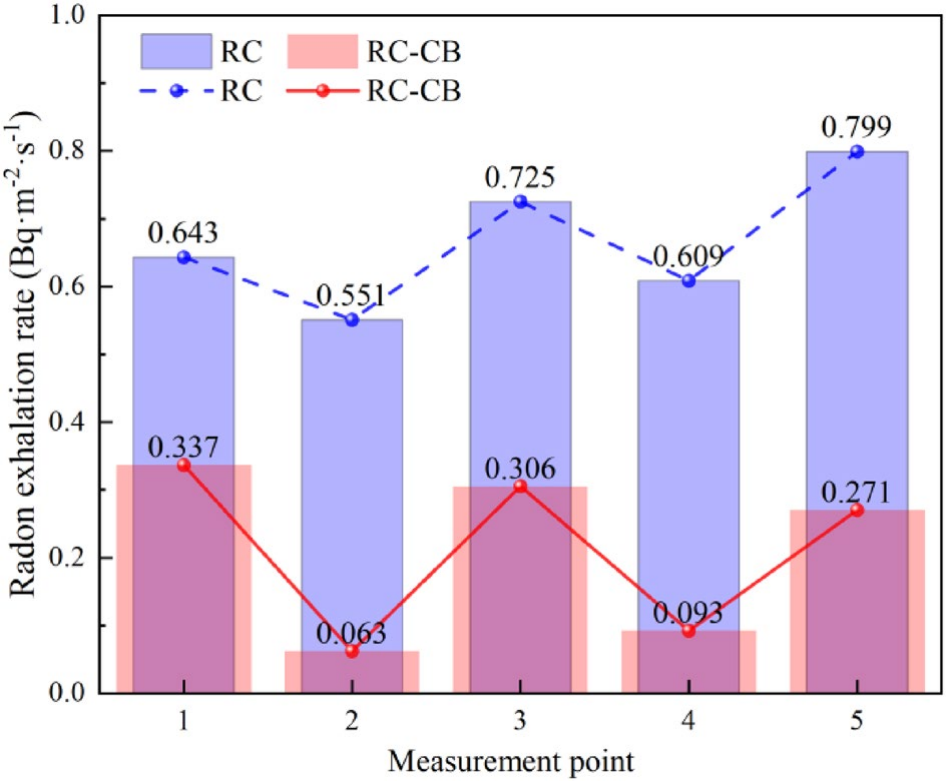
Radon exhalation rate on the beach covering layer of RC and RC-B.

Figure 4 shows the radon exhalation rates from RC and RC-B covering layers at the five measurement points. Curves of radon exhalation rate from RC and RC-B show similar trends, which indicates that the influencing mechanism of environmental conditions on radon exhalation from covering layers tends to be the same. The range for RC-B radon exhalation rate is about 0.06–0.34 Bq∙m^−2^∙s^1^ and the range for RC radon exhalation rate is 0.55–0.80 Bq∙m^−2^∙min^−1^. The difference between RC and RC-B maximum values is about 0.54 Bq∙m^−2^∙s^−1^, whereas that between the minimum values is about 0.31 Bq∙m^−2^∙s^−1^. The average radon exhalation rate from RC-B is only 0.32 times that from RC. The radon reduction effect of RC-B is much better than that of RC.

**Figure 5:**
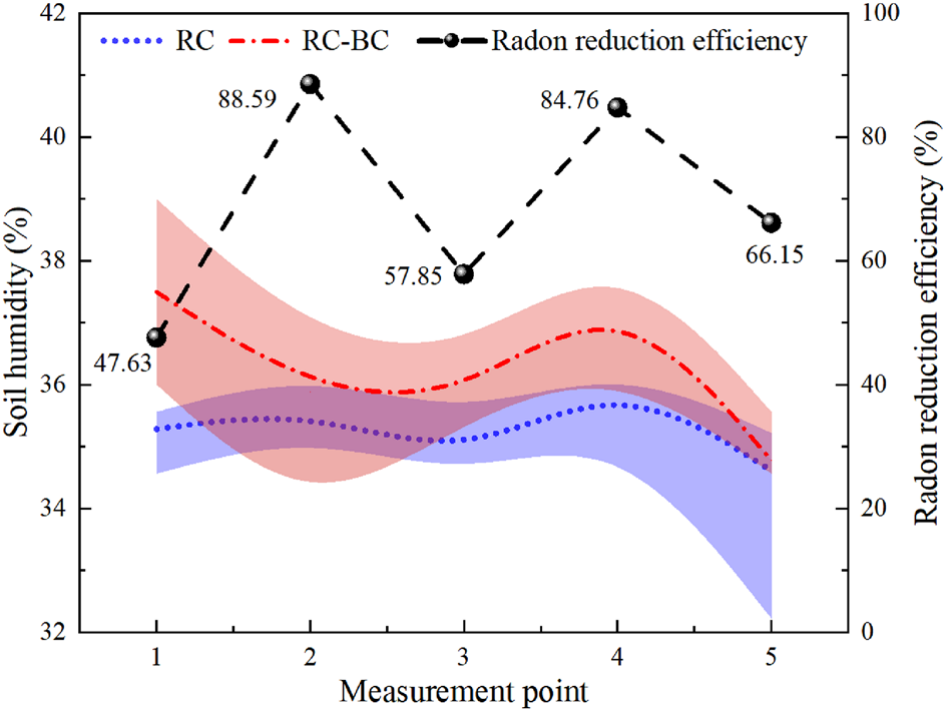
Changes in humidity and radon reduction efficiency at different measurement points.

**Figure 6:**
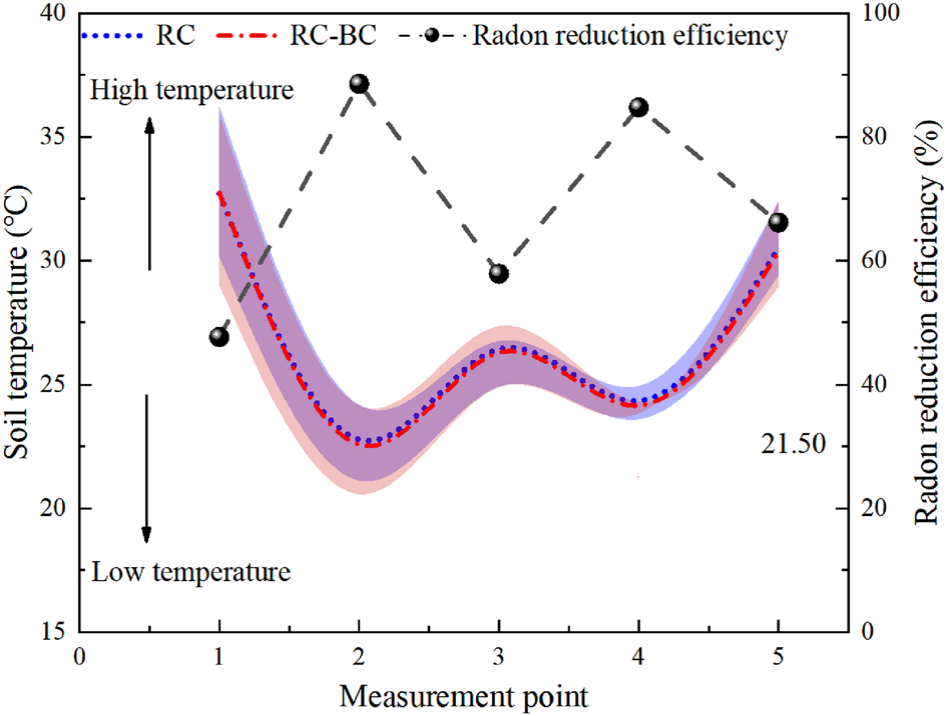
Changes in temperature and radon reduction efficiency at different measurement points.

The time-averaged value of the temperature and humidity at each measurement point is calculated and the maximum and minimum deviations are used as error bars. Figures 5 and 6 show the temperature and humidity curves in RC and RC-B measurement points.

From Figs. 4, 5 and 6, it could be seen that the radon exhalation rates from RC-B and RC have the same trend of temperature change, and the correlation is significant, but not with humidity. The humidity of the covering layer is not higher than 40%, and the moisture content of the covering layer is unsaturated. The filling effect of water on the soil pores is not obvious, and the pores of the covering layer can provide a path for radon migration^24^. The two experimental models are under the same outdoor ambient, and the evaporation effect caused by sunshine and outdoor temperature is the main power source of seepage in the covering layer^25^. Temperature enhances the seepage and diffusion of radon, which is the main reason for the variation of radon exhalations from RC and RC-B.

**Figure 7:**
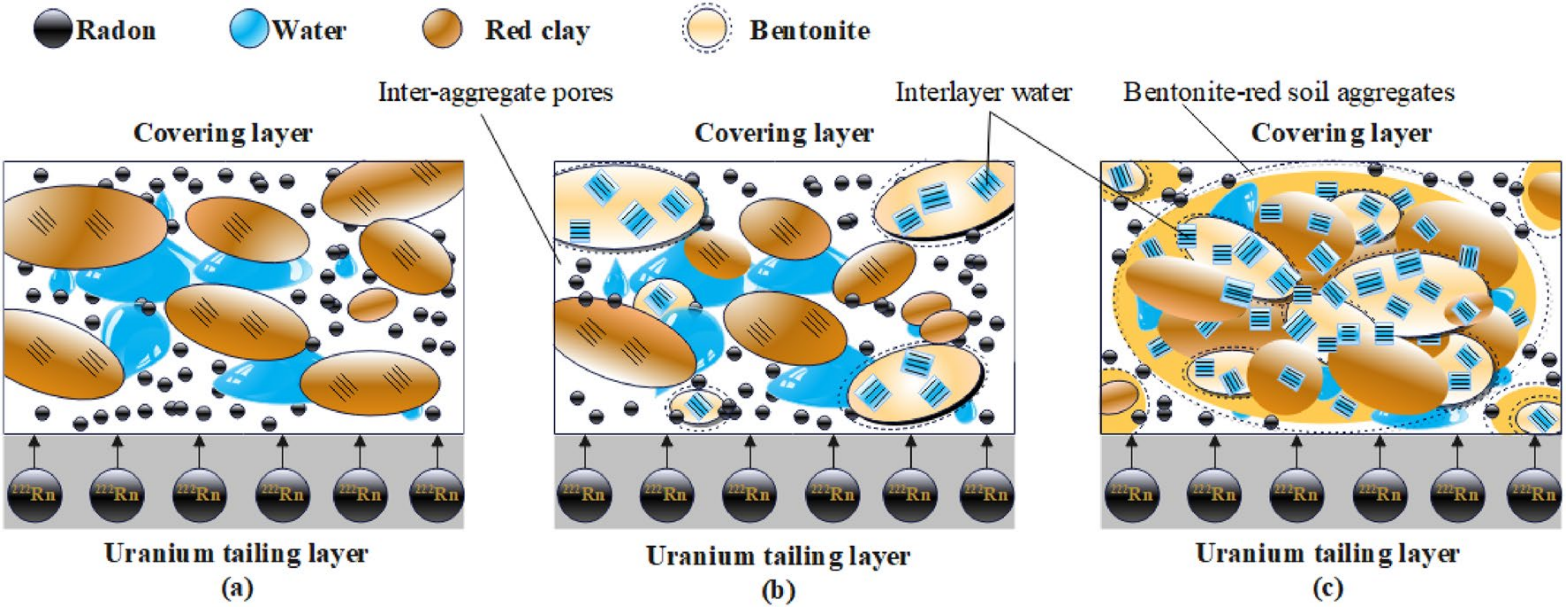
Radon control mechanism of bentonite modified covering layer. (**a**) Red clay soil covering layer, (**b**) Red clay soil-bentonite covering layer, (**c**) Red clay soil-bentonite covering layer absorbs water to form aggregates.

In bentonite, there are both intra-aggregate pores and inter-aggregate pores, forming a double pore structure^26^. As shown in Fig. 7, the pores in the polymer of bentonite adsorb a large amount of interlayer water, and this effect is not obvious in red clay soil. Bentonite would absorb water and expand with the red clay soil, forming the bentonite-red clay soil aggregates. The new aggregate would hold more water. At the macroscopic level, the soil structure shows that the bentonite-modified soil has a good water retention, small pores, and a good radon reduction effect. From Figs. 4 and 5, RC-B has the highest humidity and the lowest radon exhalation rate by comparison to those of RC.

In Fig. 6, the mean temperature values and error intervals are highly overlapping. This further validates that the addition of RC-B to the covering layer did not change the thermal insulation properties of the covering layer. The radon exhalation rates of RC-B and RC are consistent with the temperature, showing a "W" trend. This is because temperature will intensify the diffusion and seepage of radon in the cover layer, leading to an increase in radon exhalation^27^. The radon reduction efficiency shows a trend of "M", contrary to the temperature curve. This is due to the agglomeration effect of bentonite in RC-B, which reduces the pore structure of the soil and absorbs a large amount of water while in RC, the change in pore structure was relatively small. This results in a smaller effect of temperature on radon diffusion and seepage in RC-B compared to RC.

## Conclusion

1. The use of RC-B cover in uranium tailings could effectively reduce radon exhalation. Under the same outdoor conditions,the radon concentration in RC-B is lower than that in RC, and its average radon exhalation rate is only 0.32 times that in RC.
2. Bentonite could effectively change the water retention of the covering layer, but it has little effect on the insulation of the covering layer. The RC-B and RC temperature curves follow the same evolution trend and were close together in the same outdoor conditions, and the humidity curves showed a difference of 1% to 3%.
3. In the cover layer with unsaturated water content, soil water does not block the radon migration path. Soil temperature enhances the seepage and diffusion of radon, temperature was the main factor leading to the variation of RC-B and RC radon exhalation.

## Data Availability

The data from this study is available upon request from the corresponding author.
